# Fluorescent sperm offer a method for tracking the real-time success of ejaculates when they compete to fertilise eggs

**DOI:** 10.1038/srep22689

**Published:** 2016-03-04

**Authors:** Rowan A. Lymbery, W. Jason Kennington, Jonathan P. Evans

**Affiliations:** 1 Centre for Evolutionary Biology, School of Animal Biology, University of Western Australia, Crawley 6009, WA, Australia

## Abstract

Despite intensive research effort, many uncertainties remain in the field of gamete-level sexual selection, particularly in understanding how sperm from different males interact when competing for fertilisations. Here, we demonstrate the utility of broadcast spawning marine invertebrates for unravelling these mysteries, highlighting their mode of reproduction and, in some species, unusual patterns of mitochondrial inheritance. We present a method utilising both properties in the blue mussel, *Mytilus galloprovincialis*. In mytilids and many other bivalves, both sperm and egg mitochondria are inherited. We exploit this, using the vital mitochondrial dye MitoTracker, to track the success of sperm from individual males when they compete with those from rivals to fertilise eggs. We confirm that dying mitochondria has no adverse effects on *in vitro* measures of sperm motility (reflecting mitochondrial energetics) or sperm competitive fertilisation success. Therefore, we propose the technique as a powerful and logistically tractable tool for sperm competition studies. Importantly, our method allows the competitive fertilisation success of sperm from any male to be measured directly and disentangled from confounding effects of post-fertilisation embryo survival. Moreover, the mitochondrial dye has broader applications in taxa without paternal mitochondrial inheritance, for example by tracking the dynamics of competing ejaculates prior to fertilisation.

Darwin^1^ first proposed sexual selection as an evolutionary force acting on variation in reproductive success caused by (1) intrasexual competition for mates (typically among males), and (2) intersexual mate choice (typically females choosing preferred males). Since then, sexual selection has become a major focus of evolutionary and behavioural research^2^. A critical turning point in the field of sexual selection was the recognition that females often mate with multiple males, or their eggs are exposed to sperm from multiple males, meaning that sexual selection can continue after gamete release^3^. This occurs as sperm competition, where ejaculates from rival males compete for fertilisations^4^, and cryptic female choice, where females influence the outcome of such contests^5^,^6^. Both of these mechanisms of sexual selection are widespread across most sexually reproducing taxa and constitute important evolutionary forces acting on both sexes^7^,^8^,^9^. Although these processes are commonly termed ‘postcopulatory sexual selection’^10^, we prefer the term ‘gamete-level sexual selection’ to include externally fertilising animals that do not pair or copulate.

Despite intensive research on gamete-level sexual selection, there remains a taxonomic bias toward mobile, terrestrial and internally fertilising animals^3^,^11^,^12^. In particular, relatively few studies have focused on broadcast spawning marine invertebrates. Typically, these animals have sedentary or sessile lifestyles and both sexes release gametes directly into the ocean, where fertilisation occurs^13^. Although largely neglected in the context of sexual selection (but see refs 11,14), broadcast spawners exhibit several attributes that make them ideally suited for understanding gamete-level sexual selection. First, the absence of mating competition or mate choice prior to gamete release means that sexual selection operates exclusively through gamete-level interactions^11^. Second, broadcast spawners offer highly tractable systems for controlled *in vitro* experiments on gamete-level interactions. This tractability has been utilised in recent studies to characterise patterns of multivariate selection on gametes^15^,^16^, examine variation in male-female gametic and genetic compatibilities^17^,^18^, and explore the transmission of non-genetic paternal effects through sperm^19^,^20^,^21^. Finally, because broadcast spawning is likely the ancestral animal reproductive strategy^22^,^23^, the selective forces shaping this form of reproduction may yield insights into early evolutionary transitions, such as anisogamy to isogamy and external to internal fertilisation (for a recent theoretical model of this “sexual cascade” of events, see ref. 24).

One key challenge facing researchers studying gamete-level sexual selection is to determine male reproductive success at the moment of conception. Even in external fertilisers, where gamete interactions are not hidden, there are considerable logistical challenges in identifying the outcome of sperm competition at fertilisation. Sperm competitiveness has typically been estimated through paternity analyses, which involves assigning offspring parentage among two or more putative sires using genetic markers^8^. Although paternity success is clearly an important component of a male’s reproductive fitness, its use in understanding sperm competition potentially confounds variation in embryo viability with variation in fertilisation success^25^,^26^,^27^. Embryo viability can be influenced by post-competition factors such as genetic sire effects^28^,^29^, genetic compatibilities between males and females^18^,^30^,^31^, maternal allocation^32^,^33^,^34^ and non-genetic paternal effects^20^,^21^. Moreover, distinguishing between sperm competitive success on the one hand and offspring fitness on the other is crucial for evaluating whether processes such as ‘good sperm’ or ‘compatible genes’ underlie gamete-level sexual selection^26^.

Here, we propose a technique for directly examining competitive fertilisation success, using the broadcast spawning blue mussel, *Mytilus galloprovincialis* (Lamarck, 1819) as a model system. *Mytilus galloprovincialis* is a sessile, broadcast spawning bivalve with several characteristics that make it an ideal putative model system for gamete-level sexual selection. Individuals form large aggregations on intertidal substrates in temperate zones and both sexes spawn synchronously during winter months, meaning sperm and eggs from multiple individuals come into contact during each reproductive event. Moreover, unlike most animals, many bivalves (including *Mytilus* spp.) inherit mitochondrial DNA (mtDNA) from both parents in a phenomenon known as doubly uniparental inheritance (DUI)^35^. While the ultimate fate of paternal and maternal mtDNA differs depending on the sex of the offspring^36^, all embryos initially contain mitochondria transferred from the father’s sperm^37^,^38^. This presents the opportunity of labelling a male’s sperm with a vital fluorescent mitochondrial dye, allowing these sperm to compete with (undyed) sperm from other males, and tracking the real-time competitive fertilisation success of labelled sperm by counting eggs with labelled mitochondria. We develop the protocol for using this technique in evaluating sperm competition, using the mitochondria-specific vital dye MitoTracker Green FM. We test, in paired designs, whether the mitochondrial dye has any adverse effects on sperm motility and competitive fertilisation success.

## Results

### Determining the effect of mitochondrial dye on *in vitro* measures of sperm motility

We used computer-assisted sperm analyses (CASA) to evaluate sperm motility in two ejaculate samples from each of 18 males, one sample dyed with MitoTracker Green and the other left undyed. This paired experimental design therefore contrasted sperm motility between treatments while controlling for differences in ejaculate traits attributable to variation among males (see Methods). We first estimated the percentage of motile sperm from the total cell count for each sample, which was not significantly different between dyed and undyed samples (paired *t*-test, *t*_17_ = −0.62, *P* = 0.54). We estimated seven motility traits from the motile sperm: (1) average path velocity (VAP; velocity over smoothed sperm path); (2) straight-line velocity (VSL; average velocity on a straight line from start to end of path); (3) curvilinear velocity (VCL; average velocity on actual path); (4) straightness (STR; ratio of VSL to VCL); (5) linearity (LIN; ratio of VAP to VCL); (6) beat cross frequency (BCF; flagella beat rate); (7) amplitude of lateral head displacement (ALH; magnitude of sperm head displacement about the sperm trajectory). As sperm motility traits were highly correlated, we first calculated the difference in each trait between the dyed and undyed samples of each male, then reduced the set of differences in traits to principle components (PCs). Two PCs with eigenvalues >1 (collectively accounting for 88.87% of the variance in trait differences) were retained for the analysis. The first PC was loaded positively by the differences in VAP, VSL and LIN, and negatively by the difference in BCF, while the second PC was loaded positively by the differences in VCL and ALH and negatively by the difference in STR (Table 1). The means of the males’ scores for each PC (representing the composite difference between dyed and undyed measures for each male) were not significantly different from zero (PC1: *t*_17_ = 8.66 × 10^−16^, *P* = 1; PC2: *t*_17_ = 6.72 × 10^−16^, *P* = 1), thus confirming no discernible effect of the sperm dye technique on the *in vitro* measures of sperm motility.

### Competitive fertilisations

**Figure 1:**
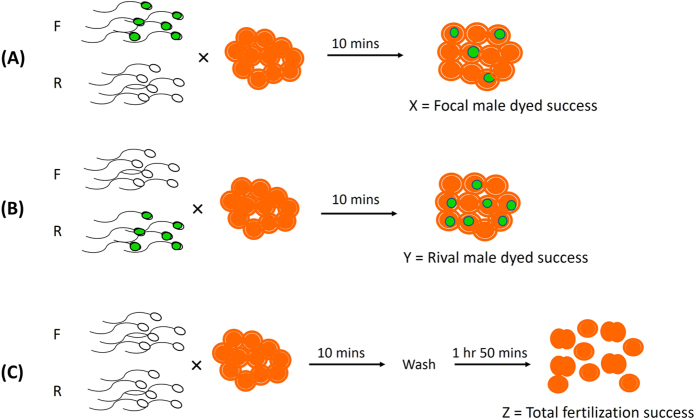
Experimental design for test of the effect of MitoTracker Green on competitive fertilisation success. Each paired comparison involved three crosses between sperm from the same two competing males (focal and rival) and eggs from the same female. (**A**) Focal male’s sperm dyed to estimate X; (**B**) rival male’s sperm dyed to estimate Y; (**C**) both males’ sperm undyed and overall fertilisation rate, Z, measured. The focal male’s dyed success, X, was then compared to his undyed success, estimated as Z-Y.

**Figure 2:**
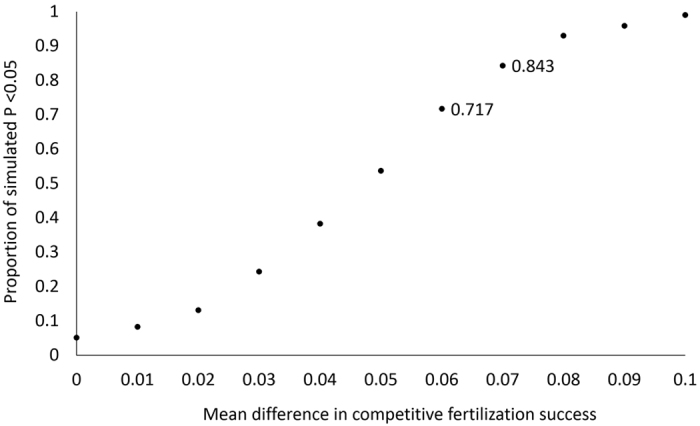
Proportion of significant simulated tests of varying differences in competitive fertilisation success between stained and unstained sperm (based on the variation and sample size in our dataset); i.e. power to detect a difference. The horizontal axis shows the varying simulated differences in the probability of success due to staining, and the vertical axis shows the proportion of significant *P* values from 1000 simulated tests. A power of 0.8 falls between a difference of 0.06 and 0.07.

To determine whether the MitoTracker dye had any effect on competitive fertilisation success, we conducted crosses with sperm from pairs of males (one termed the ‘focal’ male and the other one termed his ‘rival’) competing for the eggs of a single female (Fig. 1). For each cross (10 crosses, n = 20 males and 10 females total) we performed reciprocal trials, where one male’s sperm was dyed in each trial (i.e. focal dyed in trial A, rival dyed in trial B) to estimate the respective proportion of eggs he fertilised. We also determined the overall fertilisation rate in each cross (mean proportion of fertilised eggs 0.733 ± 0.050 s.e.m., range 0.460–0.940). We then compared the dyed success of focal males to their success when undyed (estimated by the total fertilised eggs minus the rival male’s success). There was no significant difference in the probability of successful competitive fertilisations between dyed and undyed samples (Wald *t*_17_ = 0.23, *P* = 0.821). As there were fewer replicate pairs than in the sperm motility trials, we conducted simulations to determine the power of our experimental design to detect differences in competitive fertilisation success between dyed and undyed sperm. These revealed we had 80% power to detect a difference in the proportion of fertilisation success of between 0.06 and 0.07 (Fig. 2). A mean difference of 0.06 was detected as significant in 72% of simulations, and a mean difference of 0.07 in 84% of simulations. These findings indicate that we had good power to detect small differences in fertilisation success due to the dye treatment. Indeed, in our observed data the actual mean difference in fertilisation success between dyed and undyed samples was close to zero (mean difference −0.004 ± 0.057 s.e.m.; range −0.305–0.310).

## Discussion

Our method for visualizing the real-time success of sperm when they compete to fertilise eggs offers a tractable and potentially powerful tool for studying gamete-level sexual selection. Importantly, the MitoTracker Green mitochondrial dye had no detrimental effect on sperm behaviour and had no significant influence on the capacity of sperm to fertilise eggs when in competition with rival male ejaculates. Thus, the MitoTracker dye offers an effective and reliable method for visualising the outcome of sperm competition, with important benefits for research on gamete-level sexual selection. In particular, our proposed methods overcome a major hurdle in gamete-level sexual selection research, where success in sperm competition can typically only be inferred from offspring paternity assignment.

Our experimental confirmation that the Mitotracker dye had no discernible detrimental effects on patterns of sperm motility suggests that the dye does not disrupt sperm performance or mitochondrial function. Sperm motility traits have been linked to adenosine triphosphate (ATP) production in the sperm mitochondria^39^,^40^, or to the size of the sperm midpiece where mitochondria are located^41^. Furthermore, both sperm ATP content^42^ and midpiece size^43^ can vary with the level of sperm competition. Importantly, the motility traits we measured can have fitness implications for males during competitive and non-competitive fertilisations. For example, numerous studies have reported a positive association between sperm velocity and fertilisation success across a range of taxa (reviewed in ref. 8), although there are exceptions where slower sperm have been associated with greater fertilisation benefits (e.g. see refs 44,45). In *M. galloprovincialis*, males with slower sperm that swim in more pronounced curved paths are the most successful during non-competitive fertilisation trials^15^. This may reflect their capacity to search for eggs, or the chemical attractants released by eggs^46^, in a marine environment. Given our finding that the MitoTracker Green dye had no observable effect on these specific motility traits, we propose that it may be used to assess male reproductive fitness in future studies.

Consistent with the sperm motility results, we found that the mitochondrial dye did not significantly reduce competitive fertilisation success. Although there were fewer replicate pairs for this experiment than the sperm motility trials, our analyses had the power to detect small changes (proportional change of 0.06–0.07) in fertilisation success. This further suggests that the MitoTracker dye can be applied in a sperm competition context and provides a simple and cost-effective method for assessing competitive fertilisation success, negating the need to use offspring paternity assignment as a proxy for competitive fertilisation success. This is an important methodological advance because paternity success can be influenced by a range of factors operating after fertilisation, which may or may not be related to sperm competitive ability^26^. For example, in the sea urchin *Heliocidaris erythrogramma*, variance in embryo viability and fertilisation rates are uncorrelated within male-female pairings, suggesting that fertilisation rates cannot be inferred through variance in egg hatching rates^17^. In such systems, it is critical to use techniques that can directly estimate success at the point of fertilisation, such as the MitoTracker dye.

Previous studies using fluorescent dyes to distinguish competing sperm in fertility assays have mainly focused on domestic mammals. For example, dyes have been used to visualise the number of sperm from different males bound to bovine^47^,^48^ and feline^49^ eggs. However, these prior studies could not directly determine which of the bound sperm actually achieves fertilisation. By contrast, the present technique enables us to track the real-time success of individual sperm as they fertilise eggs. As such, our proposed method offers a potentially powerful tool in the context of understanding the dynamics of sperm competition. Other studies have overcome the challenge of identifying sperm from individual males through the use of selected genetic lines that express fluorescent protein, as for example in sperm of *Drosophila melanogaster*^45^,^50^,^51^ and all cell types of *Macrostomum lignano*^52^. As with our study, these techniques make it possible to track the real-time success of sperm, although the logistical constraints of applying such methods to internal fertilisers mean that tracking sperm competition success *in vivo* is challenging in such systems. Moreover, the present technique does not require genetically modified lines for implementation, meaning that it can be applied to the sperm of any male, including those from natural populations.

The MitoTracker technique has broad applications not only across reproductive scenarios, but also potentially across taxonomic groups. The technique allows competitive fertilisation success to be tracked in any species with DUI, which include many species where knowledge about reproductive biology has potential commercial importance to fisheries^36^. Furthermore, we envisage that the mitochondrial dye technique has applications more broadly in taxa that do not have DUI of mtDNA. The mitochondrial dye could be used to track interactions of competing ejaculates *in vitro* by determining whether and how the presence of rival sperm influences pre-fertilisation performance, and examining if the capacity to influence rival sperm varies among males. For example, the use of selected lines expressing fluorescent proteins have revealed sperm displacement from the female reproductive tract by rival sperm in *M. lignano*^52^, and the adjustment of sperm swimming speed to match rival sperm in *D. melanogaster*^45^. We suggest that dyes such as MitoTracker could be used to explore such pre-fertilisation interactions in species where it is not possible to create selected genetic lines. To our knowledge, no studies have used dyes in this way to track interactions between competing sperm in an evolutionary context. We note that it may be necessary to test for the absence of an effect of dye on sperm performance in different taxa, although our expectation will be that the dye can reliably be used to stain sperm in other species. We look forward to the new insights that the implementation of this technique will bring to the field of sexual selection.

## Methods

### Sampling and spawning

We collected mussels from Cockburn, Western Australia (32°14′03.6”S, 115°76′25”E) during June to September 2014 and maintained them in aerated aquaria of recirculating seawater at the University of Western Australia until required for experiments (within 1–2 weeks of collection). Spawning was induced using a temperature increase from ambient to 28 °C^15^,^53^,^54^. Once an individual began spawning and its gender was determined, it was immediately removed from the water bath, washed in filtered seawater (FSW) to prevent contamination of gametes, placed in an individual 250 mL plastic cup and covered with FSW. Following spawning, egg densities were estimated by counting the number of eggs in a known volume under a dissecting microscope, and sperm densities were estimated from subsamples of sperm (fixed using 1% formalin) using an improved Neubauer haemocytometer. Gametes were then diluted to the concentrations required for trials (see below).

### Mitochondrial dye application

**Figure 3:**
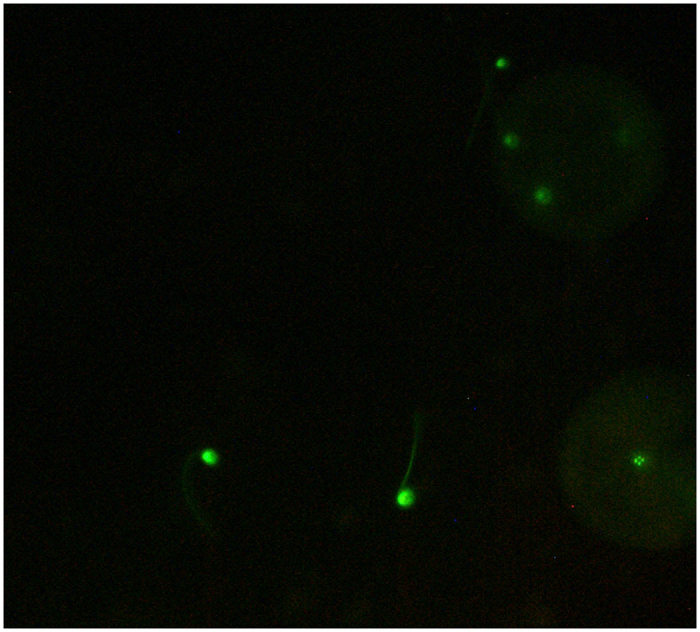
*Mytilus galloprovincialis* sperm labelled with MitoTracker Green and fertilised eggs containing mitochondria from labelled sperm. Viewed using a Zeiss Axio Imager A1 fluorescent microscope, image captured using AxioCam MRc5 and Axiovision software. Image brightness and contrast adjusted using ImageJ software.

We used the mitochondria-specific vital dye MitoTracker Green FM (Molecular Probes, Eugene, OR, USA) to stain sperm mitochondria (Fig. 3). We initially trialled a second dye colour (MitoTracker Red) but this proved to be unreliable in terms of consistency of uptake (no motile sperm were visibly labelled under fluorescence). Hence our competitive experiments involved reciprocally dying each competing male’s sperm green, rather than labelling different males’ sperm with different colours (see below). MitoTracker Green has been used previously to stain mitochondria of sperm in *Mytilus* spp. and other bivalves in order to follow paternal mitochondria through development^38^,^55^,^56^. We followed a protocol adapted from these studies for staining sperm in our experiments. All samples and solutions of dye were kept in the dark. Stock solutions of 1 mM dye were created by suspending 50 μg of MitoTracker Green in 74.5 μL dimethyl sulfoxide (DMSO). These were diluted with FSW to 10 μM working solutions. Sperm were stained in 1 mL samples (see below for sperm concentrations) containing 50 μL of working dye solution, i.e. 500 nM concentration of MitoTracker Green. Stained sperm were incubated in the dark for 10 minutes at room temperature. This was sufficient for uptake of dye by all cells in the sample (preliminary observations), while minimising sperm ageing effects, which are known to influence fertilisation rates in *M. galloprovincialis*^15^.

### Measuring sperm motility traits of dyed and undyed sperm samples

Our first experiment compared the motility (swimming characteristics) of dyed and undyed sperm samples. We prepared two 950 μL subsamples of sperm at 5 × 10^6^ sperm mL^−1^ from each of n = 18 males; 50 μL of MitoTracker working solution was added to one subsample, and 50 μL of FSW to the other. After incubation of subsamples (see above), we placed 5 μL in a 12-cell multi-test slide, previously washed with 1% polyvinyl alcohol to avoid sperm sticking to the slide. Sperm motility was characterized using computer-assisted sperm analysis (CASA; Hamilton-Thorne CEROS, Beverly, MA, USA). We used threshold values for defining static cells of 19.9 μm/s VAP and 4 μm/s for VSL. For half the males, we measured undyed samples first, while in the other half, we measured dyed samples first. A mean of 149 ± 11.7 s.e.m. motile sperm were recorded per sample. We calculated the percentage of motile sperm from the motile and total cell counts. We measured the following seven sperm motility parameters of the motile sperm, which are commonly used in studies of sperm competition and have high within-sample repeatability in *M. galloprovincialis*^15^: average path velocity (VAP), straight line velocity (VSL), curvilinear velocity (VCL), straightness (STR), linearity (LIN), beat cross frequency (BCF), the amplitude of lateral head displacement (ALH).

### Competitive fertilisation trials

To compare competitive fertilisation success of dyed and undyed sperm from the same males, we set up pairs of reciprocal competitive fertilisation trials in which sperm from the same two males, one arbitrarily chosen as the ‘focal’ male and the other as his ‘rival’, competed for fertilisation of a single female’s eggs. In these trials, 1 mL samples of sperm from each male at concentrations of 1 × 10^5^ cells mL^−1^ were added to 2 mL of eggs at 1 × 10^4^ cells mL^−1^; i.e. a final sperm:egg ratio of 10:1, shown in previous studies in this species^54^ to avoid 0% fertilisation and ceiling effects (lower variation in fertilisation rates than expected^15^,^57^). In each fertilisation, only one male’s sperm was dyed; in (A), the focal male’s sperm was dyed, and in (B) the rival male’s sperm was dyed (Fig. 1). We then estimated competitive fertilisation success of the dyed sperm in each context as the proportion of eggs containing labelled mitochondria (e.g. see labelled eggs in Fig. 3). If we denote the focal male’s dyed success as X (from trial A) and his rival’s success as Y (from trial B), we expect X = 1-Y if (i) the dye has no effect on sperm competition success, and (ii) in every trial all eggs were fertilised. We could not meet the second assumption, however, because raising sperm concentrations to levels that resulted in 100% fertilisation would have risked polyspermy, resulting in zygote failure^58^, and ceiling effects. We therefore conducted a third concurrent competitive cross (C), involving undyed sperm from the same two males to determine overall sperm fertilisation success, Z. With this estimate we set up a paired comparison in which, under a null hypothesis of no effect of dye, we expect X = Z-Y (Fig. 1). Ten paired comparisons (n = 20 males, 10 females) were used in this experiment. For further detail regarding the fertilisation procedures, see the Supplementary Methods online.

### Data analyses

Statistical analyses were carried out in R version 3.1.2^59^. For the sperm motility experiment, the percentage of motile sperm and all motility traits met the assumption of normality of differences between dyed and undyed values (Shapiro-Wilk tests, *P* > 0.05), except BCF (W = 0.89, *P* = 0.038). Measures of BCF were therefore square root transformed before performing further analyses (after transformation: W = 0.91, *P* = 0.083). The percentage of motile sperm in dyed and undyed samples was compared using a paired *t-*test. To compare the sperm motility traits of males across dyed and undyed samples, we reduced the highly correlated traits to principal components (PCs) and used PC scores in *t*-tests. Specifically, we calculated the differences between trait scores of undyed and dyed sperm samples for each male and each trait, then performed a principal component analysis on the differences using the package ‘FactoMineR’^60^ from which we retained PCs with eigenvalues >1. The PC scores for males were used as sets of differences between undyed and dyed samples and tested with one-sample *t*-tests (H_0_: μ = 0).

Dyed (X) and undyed (Z-Y) competitive fertilisation success estimates were also compared using paired analyses. Competitive fertilisation success, however, was a binomial response variable (i.e. proportions with denominator Z, the overall fertilisation rate). These data were modelled using a generalized linear mixed-effects model (GLMM) with a logit link function in the R package ‘lme4’^61^. The model was fit and parameters estimated using the Laplace approximation of the log-likelihood^62^. The model included the fixed effect of dye (i.e. dyed or undyed estimate) and a random effect for pair. The significance of the fixed effect was estimated using a Wald *t*-test, recommended by Bolker *et al*.^63^ to account for uncertainty in overdispersion estimates, because our GLMM was overdispersed (residual deviance 53.18 on 17 degrees of freedom, dispersion parameter = 3.13). Overdispersion in mixed-effects models can also be accounted for by adding an observation-level random effect, with a separate level for each individual measurement^64^. In this case, adding an observation-level random effect resulted in underdispersion (residual deviance 2.83 on 17 degrees of freedom, dispersion parameter = 0.18), but did not change the conclusions regarding the fixed effect (Wald *Z* = −0.09, *P* = 0.962, compare to test on original model in Results).

We conducted simulations to determine our power in the competitive fertilisation success experiment-i.e. the smallest difference in competitive fertilisation success between dyed and undyed samples that we could have detected with a power of 0.8 or more, given our sample size and the variation in our dataset. We provide a detailed procedure for the simulations in the Supplementary Methods online. Briefly, in each simulation we sampled 10 sets of paired values (dyed and undyed treatments) from binomial distributions in which a male’s sperm had a specified decrease in probability of fertilisation success when dyed. We then modelled these using a GLMM as described. We performed 1000 simulations for each specified difference in probability of success, and calculated the power of detecting that difference as the proportion of significant *P*-values out of 1000.

## Additional Information

**How to cite this article**: Lymbery, R. A. *et al*. Fluorescent sperm offer a method for tracking the real-time success of ejaculates when they compete to fertilise eggs. *Sci. Rep.* **6**, 22689; doi: 10.1038/srep22689 (2016).

## Supplementary Material

Supplementary Information

## Figures and Tables

**Table 1:** Sperm motility traits and principle components generated from the sets of differences in values between dyed und undyed sperm samples for each trait.

**Trait (difference between scores of undyed and dyed samples)**	**PC1**	**PC2**
VAP: average path velocity	0.94	0.28
VCL: curvilinear velocity	0.57	0.80
VSL: straight-line velocity	0.98	0.04
STR: straightness	0.50	−0.73
LIN: linearity	0.85	−0.52
ALH: amplitude of lateral head displacement	0.12	0.87
BCF: beat cross frequency*	−0.89	0.07
Eigenvalue	3.93	2.29
Cumulative per cent of variance explained	56.15	88.87

Shown are trait loadings, eigenvalues and cumulative per cent of variance in composite trait variables of the first two principle components.

^*^BCF was square root transformed prior to analyses.
