# Supplementary material for: Fluorescent sperm offer a method for tracking the real-time success of ejaculates when they compete to fertilise eggs
Source: Sci Rep. 2016 Mar 4;6:22689. doi: 10.1038/srep22689 (PMC4778040; doi:10.1038/srep22689)
Supplement: Supplementary Information [file srep22689-s1.pdf]

Fluorescent sperm offer a method for tracking the real-time success of ejaculates when they compete to fertilise eggs

Rowan A. Lymbery*, W. Jason Kennington, Jonathan P. Evans

Supplementary Methods

Experimental procedures for competitive fertilisation trials

For competitive fertilisations (A) and (B) (see Methods for experimental design), 1 mL concentrated aliquots of sperm (1×10^7 cells mL^{-1}) were made up for each male. After dying and incubation and prior to competing dyed and undyed sperm together, excess MitoTracker was removed by centrifugation in an Eppendorf MiniSpin at 1677 g (rotor radius = 6 cm, speed = 5000 rpm) for 1 minute (spinning sperm at relatively high speeds for short periods minimises oxidative stress[1,2]), removal of supernatant, and resuspension in 1 mL FSW. Importantly, the undyed samples were treated to the same centrifugation procedure as the dyed samples. Following resuspension, we adjusted sperm samples to 1mL of 1×10^5 cells mL^{-1}. The samples from the two males were then mixed together with 2 mL of eggs at 1×10^4 cells mL^{-1} (i.e. a final sperm:egg ratio of 10:1). Competitive fertilisations were allowed to proceed for 10 minutes in the dark (sperm mitochondria are clustered in the zygote and easily visible 10 minutes after fertilisation[3]). We then observed samples under a Zeiss Axio Imager A1 fluorescent microscope and assayed a haphazard sample of 100 eggs, counting the number containing dyed mitochondria.

For competitive fertilisation (C), in which no sperm were dyed, we relied on traditional methods to assay fertilisation rates, i.e. counting the proportion of eggs undergoing cleavage[4]. For these trials, we implemented strict experimental controls to ensure that fertilisation conditions (other than the absence of dyes) were similar to trials (A) and (B). Aliquots of sperm from both males were treated to the same experimental procedure as previously, including centrifugation, resuspension and dilution, and added to 2 mL of eggs at the concentrations outlined above. However, we were constrained to estimate fertilisation success 2 hours after sperm were added to the eggs, when cleavage of cells is apparent, and

not at 10 minutes as in trials (A) and (B). We therefore halted fertilisations at 10 minutes so that fertilisation rates used to estimate Z were comparable to those involving dyed sperm in each replicate. To achieve this, 10 minutes after sperm were added to the eggs, eggs were rinsed through a filter and retained in new FSW. The filter pore size was 30 µm, which retained the eggs but is many times larger than the head of *M. galloprovincialis* sperm (mean ± s.e.m. head length = 2.71 ± 0.01 µm, mean ± s.e.m. head width = 2.04 ± 0.01 µm; unpublished data from Fitzpatrick, et al.[5]). In preliminary trials, we did not detect any sperm cells remaining with retained eggs, or any visible damage to eggs. Fertilisation rates for these crosses remained high throughout the experiment. After developing in sperm-free water for a further 1 hour 50 minutes (total 2 hours), 1 mL of eggs were fixed in 10% buffered formalin until required to assess fertilisation rates. We assayed a haphazard sample of 100 eggs and calculated the proportion undergoing cleavage.

Simulation procedure for determining power to detect differences in probability of competitive fertilization with generalized linear mixed-effects model.

We used a simulation-based approach to determine the difference in probability of successful competitive fertilizations with dyed *vs*. undyed sperm that we could have detected as significant with a power of 0.8 or more, given the structure of our data. The simulations used random binomial sampling in R, which requires three inputs: the number of samples ('n'), the number of trials in each sample ('size'), and the probability of success for each trial ('prob'). The output is number of successes in each sample. First, we simulated the number of undyed successful competitive fertilizations for each focal male, where 'n' = 10 focal males, and the 'size' input was the vector of actual number of trials for each focal male (corresponding to the total number of eggs fertilized across both competitors, Z; see Methods). The input for 'prob' was a vector of proportion of actual undyed competitive fertilizations [(Z-Y)/Z; see Methods] for each focal male, i.e. we used the observed undyed competitive sperm success of focal males to set their 'baseline' probability of success in random sampling. The random binomial sampling then used this baseline probability to provide a simulated number of undyed successful competitive fertilizations for each focal male.

We then simulated the number of dyed successful competitive fertilizations in a similar way. The inputs for 'n' and 'size' were the same as above. The input for 'prob' again used the vector of 'baseline' probabilities, but subtracted a constant effect on probability due to dye that we wanted to simulate. For example, when simulating a decrease in the probability of success due to dye of 0.05 (5%), 'prob' was the vector of baseline probabilities minus 0.05. In this way we were able to simulate paired values of undyed and dyed success for each male. We combined the simulated undyed and dyed data, and assigned the factors 'Treatment' (dyed or undyed) and 'Pair'. We then ran a generalized linear mixed model on the simulated data (see Methods) and stored the P-value for the fixed effect 'Treatment'. The entire simulation procedure was repeated 1000 times for a particular simulated effect of dye, and the power to detect the simulated effect estimated by the proportion of P-values that were statistically significant (<0.05).

References

1 Shekarriz, M., DeWire, D. M., Thomas Jr, A. J. & Agarwal, A. A method of human semen centrifugation to minimize the iatrogenic sperm injuries caused by reactive oxygen species. *European Urology* **28**, 31-31 (1995).

2 Carvajal, G. *et al.* Effects of centrifugation before freezing on boar sperm cryosurvival. *J. Androl.* **25**, 389-396 (2004).

3 Obata, M. & Komaru, A. Specific location of sperm mitochondria in mussel *Mytilus galloprovincialis* zygotes stained by MitoTracker. *Dev. Growth Diff.* **47**, 255-263 (2005).

4 Marshall, D. J., Styan, C. A. & Keough, M. J. Intraspecific co-variation between egg and body size affects fertilisation kinetics of free-spawning marine invertebrates. *Mar. Ecol. Prog. Ser.* **195**, 305-309 (2000).

5 Fitzpatrick, J. L., Simmons, L. W. & Evans, J. P. Complex patterns of multivariate selection on the ejaculate of a broadcast spawning marine invertebrate. *Evolution* **66**, 2451-2460 (2012).
